# Anesthetic management of coronary artery reconstruction in a patient with myocardial ischemia caused by an anomalous origin of the right coronary artery running between the great vessels: a case report

**DOI:** 10.1186/s40981-025-00786-9

**Published:** 2025-04-17

**Authors:** Riki Kuzuno, Shuji Kawamoto, Kenichiro Tatsumi, Chikashi Takeda, Moritoki Egi

**Affiliations:** Department of Anesthesia, Kyoto University Hospital, 54, Shogoinkawahara-Cho, Sakyo-Ku, Kyoto, 606 - 8507 Japan

**Keywords:** Anomalous coronary artery originating from the opposite sinus of Valsalva, Coronary artery anomaly, Sudden cardiac death, Coronary unroofing, R-ACAOS

## Abstract

**Background:**

Coronary artery origin anomalies, though often incidentally detected, can lead to sudden death. Comprehensive perioperative management is essential. We report a case of an anomalous right coronary artery (RCA) arising from the left main coronary artery (LMCA) and coursing between the aorta and pulmonary artery, discovered after myocardial infarction, in which intraoperative management ensured successful coronary reconstruction.

**Case presentation:**

A 49-year-old woman presented with chest pain and ST segment elevation. Coronary angiography revealed an anomalous RCA demonstrating compressive ischemia by the aorta and pulmonary artery. Preoperatively, blood pressure was stabilized with an isosorbide dinitrate patch. Under cardiopulmonary bypass, the RCA was transected and reanastomosed to its physiological aortic position. Intraoperatively, nicorandil was administered to suppress vascular smooth muscle contraction, while five-lead ECG, transesophageal echocardiography, and operative ultrasound monitoring enabled early detection of ischemia and prevented hypertension. Postoperative ventricular premature contractions resolved by the next day, with uneventful recovery.

**Conclusions:**

Targeted pharmacologic blood pressure control and multimodal monitoring are vital for safe perioperative outcomes in anomalous coronary artery origin cases.

## Background

Coronary artery origin anomalies are often asymptomatic and detected incidentally, but these anomalies can be life-threatening and are responsible for a significant proportion of sudden cardiac events, particularly in young athletes [1, 2]. Since this condition is rare, reports of surgical interventions are limited in the medical literature. In this report, we describe a case of myocardial ischemia resulting from compression of the right coronary artery (RCA), which originated from the left main coronary artery (LMCA) between the aorta and pulmonary artery. The patient was managed with coronary artery reconstruction to address the ischemia and prevent further complications.

## Case presentation

A 49-year-old woman presented at a local clinic with chest pain that occurred while washing her hair. An electrocardiogram (ECG) revealed ST elevation in leads II, III, and aVF, prompting referral to our hospital. Her medical history included gastric cancer surgery, but she had no history of smoking or alcohol consumption. Upon arrival, her chest pain had resolved, and her vital signs included blood pressure (BP) of 147/97 mmHg and heart rate of 102 beats per minute.

**Fig. 1:**
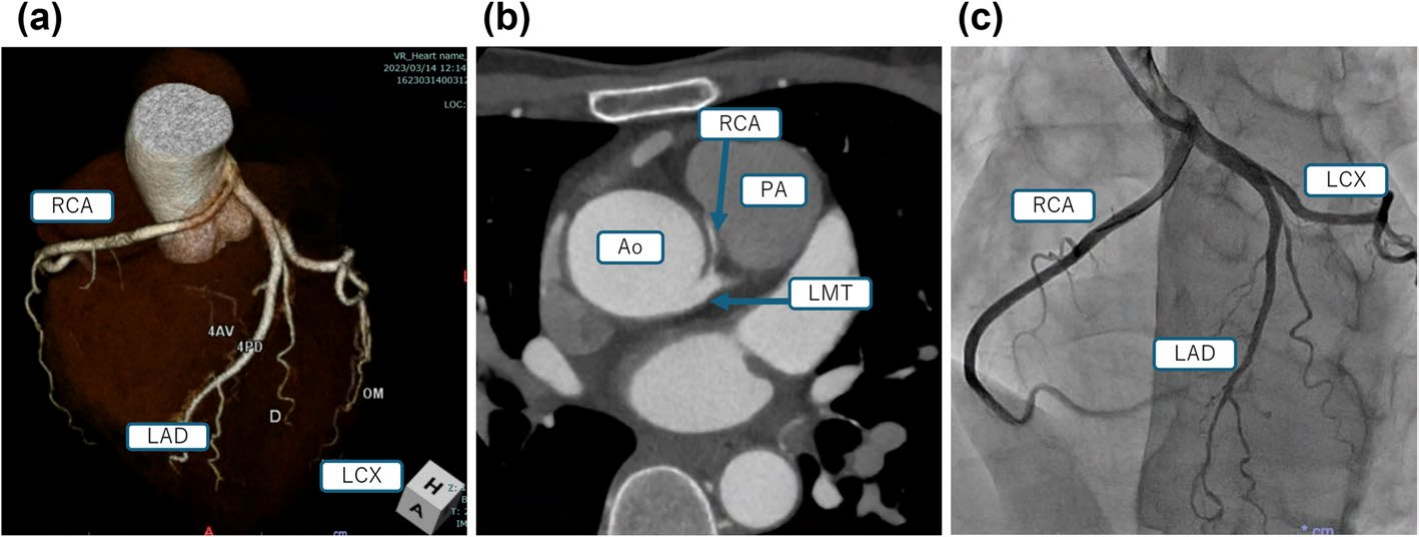
The right coronary artery originated from the left main coronary artery. **a**, **b** Coronary computed tomography angiography. **c** Coronary angiography. RCA, right coronary artery; LAD, left anterior descending artery; LCX, left circumflex artery; LMT, left main trunk; Ao, aorta; PA, pulmonary artery

Shortly after arrival at hospital, BP was 141/90 mmHg, and ST elevation had improved. Laboratory tests revealed elevated markers of myocardial injury: CK 397 U/L, CK-MB 11 U/L, and troponin T 0.199 ng/ml (ECLIA). Transthoracic echocardiography (TTE) showed a preserved left ventricular ejection fraction (LVEF) of 64%, with no evidence of valvular disease, cardiomegaly, or wall motion abnormalities. Coronary computed tomography angiography (CTA) revealed that the RCA originated from the LMCA and coursed between the ascending aorta and pulmonary artery, consistent with an anomalous coronary artery originating from the opposite sinus of Valsalva (ACAOS) (Fig. 1a, b). Coronary angiography (CAG) confirmed this right ACAOS without significant stenosis (Fig. 1c). Intravascular ultrasound (IVUS) revealed a flattened RCA origin, suggesting external compression. Elevated BP or smooth muscle contraction within the aortic wall may contribute to ischemia by compressing the RCA [3], and this may also have been the mechanism in this case.

Since CAG showed adequate coronary flow upon admission, emergency surgery was deferred in favor of BP control and further evaluation. Laboratory tests on the following day showed that the CK level had peaked, further supporting conservative management. In preoperative management, nifedipine 20 mg/day was initiated on the first day of admission. Since the patient experienced intermittent chest pain at night, the dose was increased to 40 mg/day. BP was around 120–130 mmHg on the following day. Chest pain improved on the next night, but the patient had some chest discomfort. On day 3, a 4-mg isosorbide dinitrate patch was started. Continuous intravenous infusion of nicorandil was also initiated at a rate of 2 mg/h but was discontinued after 8 h due to reflex tachycardia. At this time, BP had decreased to 90–110 mmHg, and heart rate had increased from 80 to 150 bpm. On day 4, although chest symptoms had subsided, reflex tachycardia persisted, prompting a switch from nifedipine to amlodipine 5 mg/day. On day 5, orthostatic hypotension occurred, with BP dropping to 80 mmHg, and amlodipine was subsequently discontinued. Thereafter, only the isosorbide dinitrate patch was maintained until the day of surgery, and there were no further symptoms or hypotension. BP stabilized at around 100 mmHg with no recurrence of chest pain after day 2. Cardiac magnetic resonance imaging (MRI) revealed apical thinning, suggesting previous ischemic episodes, and surgery was scheduled for day 14.

During surgery, in addition to standard cardiac anesthetic management, we focused on preventing hypertension, avoiding sympathetic stimulation, and closely monitoring cardiac function. Given the BP of 147 mmHg at symptom onset and the observed correlation between a decrease in daytime BP below 120 mmHg to around 100 mmHg and resolution of nocturnal chest pain, we set a target BP of around 100 mmHg, with an upper limit of 120 mmHg. To achieve this, continuous intravenous infusion of nicorandil at a rate of 4 mg/h, which is known to suppress smooth muscle contraction, was administered with particular caution due to the previous reflex tachycardia during preoperative management. For pain management, 250 μg of fentanyl was administered before the incision, and remifentanil was continuously infused at 0.2 μg/kg/min to avoid sympathetic stimulation caused by surgical stress. As a result, while BP briefly rose to about 140 mmHg during positional changes and initiation of surgery, it quickly returned to target levels, staying near 100-mmHg systolic for most of the pre-reconstruction period. No ischemic changes, such as ST segment elevation, were observed on the ECG during the operation.

Real-time monitoring of RCA blood flow was performed using transesophageal echocardiography (TEE). During coronary artery reconstruction, the RCA origin was found to be embedded within the aortic wall. Part of the origin was unroofed, and the RCA was repositioned and fixed at an anatomically appropriate location. Post-reconstruction monitoring with TEE and operative field ultrasonography confirmed satisfactory RCA blood flow, measured at 70 mL/min.

**Fig. 2:**
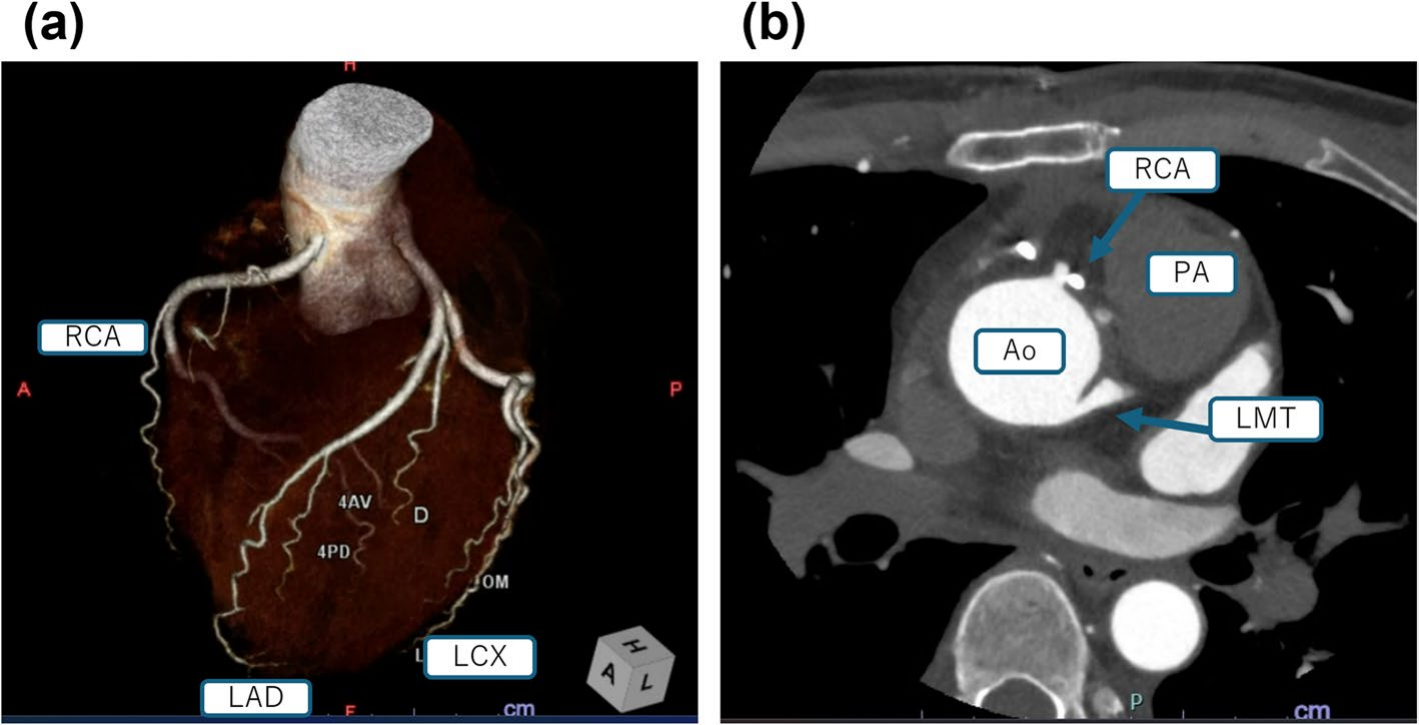
Postoperative coronary CTA findings. **a**, **b** The coronary artery was reimplanted in its normal position. Imaging showed good patency. RCA, right coronary artery; LAD, left anterior descending artery; LCX, left circumflex artery; LMT, left main trunk; Ao, aorta; PA, pulmonary artery

The total anesthesia time was 346 min, the operation time was 242 min, and the cardiopulmonary bypass time was 110 min. Blood loss was 260 mL, and the surgery was completed as planned. The patient was transferred to the ICU postoperatively and extubated the following morning. Following restoration of cardiac rhythm, intermittent ventricular premature contractions persisted until the day after surgery, but these contractions resolved completely thereafter. Follow-up TTE 1 week postoperatively revealed no abnormalities, with an LVEF of 73%. CTA confirmed that the RCA was patent in its reconstructed position (Fig. 2). The patient experienced no complications and was discharged uneventfully on postoperative day 15.

## Discussion

In this case, myocardial ischemia was considered to have resulted from compression of the RCA, which originated from the LMCA and passed between the aorta and pulmonary artery. Coronary artery reconstruction was performed under strict blood pressure control to minimize sympathetic stimulation and inhibit smooth muscle contraction.

ACAOS occurs in approximately 0.44% of the population and is often asymptomatic [1, 4]. Because many individuals remain asymptomatic, opportunities for cardiovascular imaging are limited, which may in turn restrict the accuracy of prevalence estimates. Moreover, ACAOS encompasses a variety of subtypes, and no definitive treatment strategy has been established. However, in certain subtypes (59.1%), such as that in which the coronary artery courses between the aorta and pulmonary artery, the risk of sudden cardiac death is significantly elevated [4]. ACAOS may account for up to 19% of sudden cardiac deaths in young athletes, underscoring the importance of accurate diagnosis and appropriate treatment [1, 2]. There are some case reports from a cardiac surgery perspective that discuss surgical procedure selection and preoperative evaluation but few reports with detailed discussions of preoperative and anesthetic management. In our case, appropriate surgical management, including unroofing and reimplantation of the coronary artery, was successfully performed.

Recent studies suggest that when the coronary artery passes between the aorta and pulmonary artery, it often runs intramurally within the aortic wall. Sympathetic stimulation can induce smooth muscle contraction in the aortic wall, leading to increased intramural pressure and resulting in ischemia. As described by Bartoli et al. [3], myocardial ischemia occurs when dobutamine and phenylephrine are administered to calves with ACAOS, a congenital anomaly of the coronary arteries. While this poses a lower risk than left ACAOS that runs between the great vessels, right ACAOS also carries a high risk of sudden death [1, 5]. Physiological stenosis due to hypoplasia of the coronary origin has been proposed as another potential mechanism [5, 6]. In this case, coronary CTA revealed an acute bifurcation angle of about 10°, and IVUS showed stenosis around the origin, both of which indicated a high risk of ischemia associated with intramural coronary involvement. Based on these findings, the patient’s symptoms were considered to have occurred against the background of age-related enlargement of major vessels and arterial wall stiffening, triggered by transient blood pressure elevation and sympathetic stimulation. Given that the symptoms appeared while washing their hair and did not appear again after blood pressure was controlled, we assumed that avoiding sympathetic stimulation and hypertension was essential approaches in perioperative management.

Preoperative management in the current case focused on avoiding hypertension and minimizing sympathetic stimulation. Although the long-term treatment strategy remains uncertain due to the diverse mechanisms of myocardial ischemia and limited evidence supporting each intervention, surgical revascularization is generally recommended. Conservative therapies, such as beta-blocker administration and exercise restriction, are not strongly advocated [1, 7, 8]. There are few detailed reports that delve into specific preoperative management. In our case, we aimed to reduce sympathetic stimulation by ensuring strict bed rest and applying isosorbide dinitrate patches to suppress vascular smooth muscle contraction. Moreover, to prevent hypertension during symptomatic episodes and maintain resting blood pressure, use of calcium channel blockers was considered. Given the short intramural segment of the coronary artery, we opted for a procedure that involved unroofing the intramural segment and anatomically reimplanting the RCA in its original position. Although coronary artery bypass grafting (CABG) carries a lower risk of aortic valve injury compared to reimplantation [9], it was not chosen due to the lower long-term patency rates reported for bypass grafts in ACAOS cases [10].

During anesthetic management, systolic BP was controlled to prevent exacerbation of ischemia. Given the systolic BP of 147 mmHg at symptom onset and the observation that nocturnal chest pain resolved when resting systolic BP decreased to below 120 mmHg and stabilized around 100 mmHg, the target systolic BP was set at about 120 mmHg. Preoperative isosorbide dinitrate and intraoperative nicorandil were administered to suppress vascular smooth muscle contraction. Real-time evaluation with TEE, operative field ultrasound, and 5-lead ECG monitoring enabled prompt detection of any abnormalities. As a result, there was no aortic valve injury, the reconstructed coronary flow remained stable, and the postoperative course was uneventful. Postoperatively, transthoracic echocardiography and coronary CTA were performed once the patient’s condition stabilized, and these procedures confirmed adequate coronary flow and the absence of wall motion abnormalities prior to discharge.

This case shows that preoperative anatomical assessment using imaging studies and establishment of individualized management targets based on the patient’s symptoms can contribute to successful treatment outcomes in ACAOS.

## Data Availability

Data for this case report are unavailable for public access because of patient privacy concerns.

## Abbreviations

RCA: Right coronary artery
LMCA: Left main coronary artery
ECG: Electrocardiogram
BP: Blood pressure
TTE: Transthoracic echocardiography
LVEF: Left ventricular ejection fraction
CTA: Computed tomography angiography
ACAOS: Anomalous coronary artery originating from the opposite sinus of Valsalva
IVUS: Intravascular ultrasound
